# Tuning the cache memory usage in tomographic reconstruction on standard computers with Advanced Vector eXtensions (AVX)

**DOI:** 10.1016/j.dib.2014.12.010

**Published:** 2015-01-08

**Authors:** Jose-Ignacio Agulleiro, Jose-Jesus Fernandez

**Affiliations:** a Dep. Informatica, Universidad de Almeria, 04120 Almeria, Spain; b Centro Nacional de Biotecnologia (CNB-CSIC), Campus Cantoblanco, 28049 Madrid, Spain

## Abstract

Cache blocking is a technique widely used in scientific computing to minimize the exchange of information with main memory by reusing the data kept in cache memory. In tomographic reconstruction on standard computers using vector instructions, cache blocking turns out to be central to optimize performance. To this end, sinograms of the tilt-series and slices of the volumes to be reconstructed have to be divided into small blocks that fit into the different levels of cache memory. The code is then reorganized so as to operate with a block as much as possible before proceeding with another one. This data article is related to the research article titled **Tomo3D 2.0 – Exploitation of Advanced Vector eXtensions (AVX) for 3D reconstruction** (Agulleiro and Fernandez, 2015) [1]. Here we present data of a thorough study of the performance of tomographic reconstruction by varying cache block sizes, which allows derivation of expressions for their automatic quasi-optimal tuning.

**Specifications table**

**Table:** 

Subject area	*Computer Science*; *Scientific computing*
More specific subject area	*High performance computing*; *Electron tomography*
Type of data	*Graph, figure*
How data was acquired	*Processing time measured in the computer*
Data format	*Analyzed output data*
Experimental factors	*Tilt-series of 140 images of 2048*×*2048 and 4096*×*4096 pixels were used as inputs.*
Experimental features	*Tomograms of 2048*×*2048*×*256 and 4096*×*4096*×*256 voxels were reconstructed from the input tilt-series with 15 iterations of the iterative reconstruction method SIRT and varying the usage of the first (L1) and last (LLC) level of cache memory.*
Data source location	*Computational infrastructures at the High Performance Computing and Algorithms group, University of Almeria, Almeria, Spain.*
Data accessibility	*Data are with this article.*

## Value of the data

• An analysis of the influence of the cache blocking in the performance of tomographic reconstruction is presented.
• The study allows derivation of formulas for quasi-optimal values of block sizes at different levels of cache memory.
• The data are useful to properly tune the cache memory usage for fast tomographic reconstruction on standard computers.

## Data, experimental design, materials and methods

**Fig. 1:**
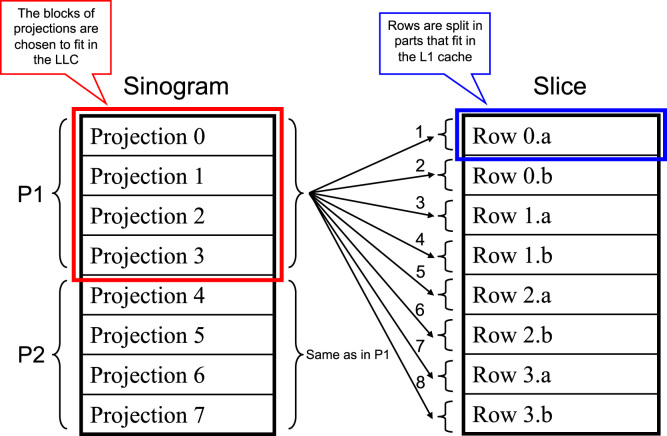
The cache mechanism implemented in Tomo3D 2.0 to efficiently access the image data using the AVX extensions. Sinograms are divided into blocks which fit in the LLC, while every row in a slice is broken as many times as necessary to fit in the L1 cache. Here an illustrative example with a sinogram of 8 projections and a slice of 4 rows is shown. The sinogram has been divided into two blocks of projections (P1 and P2) and each row of the slice has been broken in two parts (‘a’ and ‘b’). Each part of a row is processed with all projections in the current block P1 before switching to another part, that is, row 0.a with projection 0, 1, 2, 3, row 0.b with projection 0, 1, 2, 3, row 1.a with projection 0, 1, 2, 3, and so forth. Once all projections in P1 have been processed, the same procedure is repeated with P2.

Tomographic reconstruction implemented in Tomo3D 2.0 includes a new blocking mechanism to take advantage of the processor cache and reduce cache misses (Fig. 1). Slices and sinograms are divided into small blocks that fit into the different levels of cache memory. The blocks in cache memories are re-used as much as possible before proceeding with others, thus minimizing the data exchange with main memory. Cache blocking turns out to be of paramount importance to maximize performance of tomographic reconstruction with Advanced Vector eXtensions (AVX), where sets of eight slices of the volume are reconstructed simultaneously thanks to these vector instructions [1].

As shown in Fig. 1, our new cache mechanism takes advantage of both the first (L1) and the last (LLC) level of cache. On the one hand, sinograms are divided into blocks of projections whose size is chosen to fit in the LLC. On the other hand, the different rows of a slice are broken in smaller parts that fit in the L1 cache, depending upon an integer split factor denoted by ‘splitf’. A part of a row is then kept in L1 while being processed with all projections in the current block of projections, which in turn is kept in the LLC, hence maximizing the use of cache memory. This splitting of sinograms and slices is applied to the Forward and Backward projection steps of the SIRT iterative reconstruction algorithm [1]. To evaluate this cache blocking mechanism, we carried out a thorough study of the performance by varying the block sizes for the LLC and L1 cache memories. The results are reported in the following section.

For the evaluation, we used two platforms based on the Sandy Bridge Intel microarchitecture. The first one, referred to as ‘Platform 1’, was a standard desktop computer with an Intel Core i7-2600 (quad core) at 3.4 GHz, with 32 kB of L1 cache per core and 8 MB of LLC (third level of cache, shared by the four cores). The second platform, ‘Platform 2’, was a node of a cluster. It had two Intel Xeon E5-2650 (octo core) at 2 GHz, with a total of 16 cores, with 32 kB of L1 cache per core and 20 MB of LLC per CPU (i.e. shared by the eight cores). We used datasets of representative sizes of current structural studies by electron tomography. Thus, we selected tilt-series of 140 images of sizes 2048×2048 and 4096×4096 pixels, in the tilt range [−70°, 69°], to generate reconstructed volumes of 2048×2048×256 and 4096×4096×256 voxels, respectively. In the following, they are denoted by 2K and 4K datasets.

The cache mechanism is included in Tomo3D 2.0 [1], which was compiled with the Intel C/C++ Compiler and was run under Linux. The evaluation was based on 15 iterations of SIRT using AVX instructions (i.e. eight slices reconstructed simultaneously) and all combinations of platforms and datasets were covered. To perform a more general analysis, we evaluated two situations. Firstly, threads were created to use all cores available in a chip (denoted by 4*T* in Platform 1 and 8*T* in Platform 2); secondly, only half (2*T* and 4*T*, respectively). All the experiments were carried out five times, and the average computation times were then calculated.

## Tuning the cache memory usage

We conducted a set of experiments aiming at analyzing the influence of our strategy to exploit cache memory and determining the optimal block sizes for the L1 and LLC caches. For each L1 block size, the LLC block size was varied and the execution time was measured.

**Fig. 2:**
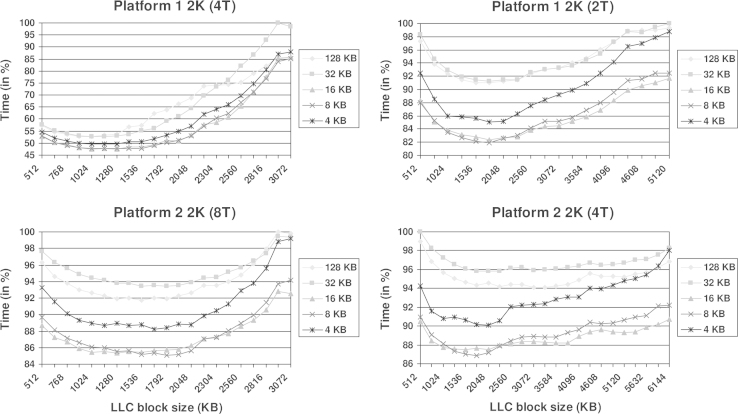
Performance of the AVX implementation of tomographic reconstruction as a function of the L1 and LLC cache block sizes obtained with the 2K dataset.

**Fig. 3:**
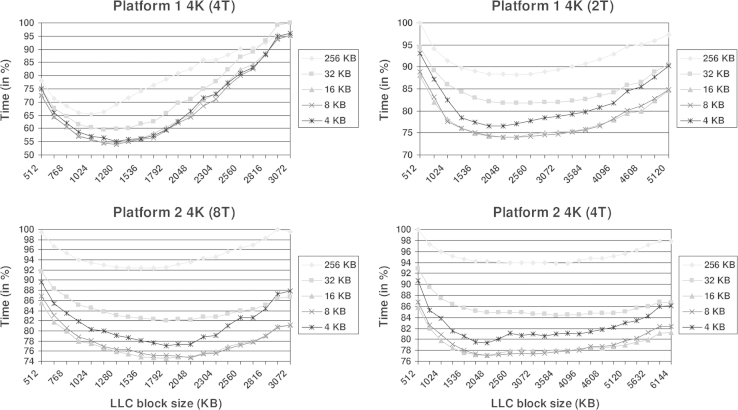
Performance of the AVX implementation of tomographic reconstruction as a function of the L1 and LLC cache block sizes obtained with the 4K dataset.

Figs. 2 and 3 show the processing time obtained with the 2K and 4K datasets, respectively. The time is represented in % with regard to the slowest one in each plot. The results from Platform 1 are shown on the top whereas those from Platform 2 are on the bottom. The results from the use of all or half of cores in a chip are presented on the left and right columns, respectively. The plots include a curve for the L1 block size corresponding to the native row size of a slice, which is equivalent to a ‘splitf’ of 1. This curve is 128 kB for the 2K dataset and 256 kB for the 4K one (i.e. as many vectors of eight components as the row size, and also adding those for the symmetric pixels, using 32-bit floating point numbers). Furthermore, the plots include curves for L1 block sizes of 32–4 kB. These represent ‘splitf’ factors of 4, 8, 16 and 32 for the 2K dataset and 8, 16, 32 and 64 for the 4K dataset. Note that 32 kB is the size of the L1 cache available in each core.

The plots in Figs. 2 and 3 tend to cup-like shapes that demonstrate that the cache block sizes may have a striking influence on the performance. As shown in the figures, the reduction of the processing time may be as dramatic as around 50% with respect to the poorest case (see top left panels). Therefore, tuning of the cache block sizes is paramount for an optimal execution. In case not all cores are to be used, there is a wide range of values that turns out to be optimal for the LLC block size, as pointed by the flatter performance curves (see right column in Figs. 2 and 3). If all cores are used, the optimal values concentrate in a narrower range. We have found that the formula LLC_size/(1.6×*T*), with LLC_size denoting the size of the LLC cache memory and *T* referring to the number of running threads, provides a good generic value for the LLC block size. As far as the L1 block size is concerned, the plots clearly show that the rows of the slices have to be broken into smaller rows that should take up to half the L1 size. Values of 8 kB and 16 kB turn out to be optimal whereas lower values (4 kB) do not allow full advantage of the cache memory. If the block is set to the whole L1 cache memory (32 kB), the effect may still be beneficial for large problem sizes (4K dataset, Fig. 3), when compared to the behaviour of the native row size. However, it may be negligible or unfavourable for smaller problem sizes (2K dataset, Fig. 2), due to the penalty derived from the increased number of iterations associated with the corresponding loop of the algorithm [1].

In conclusion, and based on these results, the formulas for quasi-optimal values of the LLC and L1 block sizes in AVX-vectorized and multithreaded tomographic reconstruction are given by LLC_size/(1.6×*T*) and L1_size/2, respectively, with LLC_size and L1_size denoting the size of the cache memory at that level and *T* referring to the number of running threads.

## References

[1] Jose-Ignacio Agulleiro, Jose-Jesus Fernandez (2015). Tomo3D 2.0 – Exploitation of Advanced Vector eXtensions (AVX) for 3D reconstruction. J. Struct. Biol..

